# Measuring the Phytochemical Richness of Meat: Effects of Grass/Grain Finishing Systems and Grapeseed Extract Supplementation on the Fatty Acid and Phytochemical Content of Beef

**DOI:** 10.3390/foods12193547

**Published:** 2023-09-24

**Authors:** Lucas Krusinski, Isabella C. F. Maciel, Stephan van Vliet, Muhammad Ahsin, Guanqi Lu, Jason E. Rowntree, Jenifer I. Fenton

**Affiliations:** 1 Department of Food Science and Human Nutrition, Michigan State University, East Lansing, MI 48824, USA; krusins1@msu.edu; 2 Department of Animal Science, Michigan State University, East Lansing, MI 48824, USA; isabellafmaciel@gmail.com (I.C.F.M.); rowntre1@msu.edu (J.E.R.); 3 Center for Human Nutrition Studies, Department of Nutrition, Dietetics, and Food Sciences, Utah State University, Logan, UT 84322, USA; stephan.vanvliet@usu.edu (S.v.V.); a02387561@usu.edu (M.A.); 4 Department of Epidemiology and Biostatistics, Michigan State University, East Lansing, MI 48824, USA; luguanqi@msu.edu

**Keywords:** cattle, pasture, grapeseed extract, beef, fatty acids, metabolomics, phenols, phytochemicals

## Abstract

Grass-finished beef (GFB) can provide beneficial bioactive compounds to healthy diets, including omega-3 polyunsaturated fatty acids (*n*-3 PUFAs), conjugated linoleic acid (CLA), and secondary bioactive compounds, such as phytochemicals. The objective of this study was to compare fatty acids (FAs), micronutrients, and phytochemicals of beef fed a biodiverse pasture (GRASS), a total mixed ration (GRAIN), or a total mixed ration with 5% grapeseed extract (GRAPE). This was a two-year study involving fifty-four Red Angus steers (*n* = 54). GFB contained higher levels of *n*-3 PUFAs, vitamin E, iron, zinc, stachydrine, hippuric acid, citric acid, and succinic acid than beef from GRAIN and GRAPE (*p* < 0.001 for all). No differences were observed in quantified phytochemicals between beef from GRAIN and GRAPE (*p* > 0.05). Random forest analysis indicated that phytochemical and FA composition of meat can predict cattle diets with a degree of certainty, especially for GFB (5.6% class error). In conclusion, these results indicate that GFB contains higher levels of potentially beneficial bioactive compounds, such as *n*-3 PUFAs, micronutrients, and phytochemicals, compared to grain-finished beef. Additionally, the *n*-6:*n*-3 ratio was the most crucial factor capable of separating beef based on finishing diets.

## 1. Introduction

Health organizations recommend reducing red meat consumption for human health and environmental reasons [1]. Beef is often associated with various metabolic diseases, and its production is thought to contribute to climate change [2]. While there are legitimate concerns about the current beef production, putting all beef in the same category may be reductionistic [3]. Cattle management practices need to be considered when nutritional claims are made. For example, grass-finished beef (GFB) produced in agroecological ways generally aligns with the preferences of consumers who are worried about both their well-being and environmental impact [4]. GFB is an important alternative contributing to food sustainability goals by providing higher amounts of potentially beneficial nutrients [5]. First, GFB contains more omega-3 (*n*-3) polyunsaturated fatty acids (PUFAs), more conjugated linoleic acid (CLA), less omega-6 (*n*-6) PUFAs, and less cholesterol-raising saturated fatty acids (SFAs) than conventional grain-finished beef [6,7,8,9]. Second, other health-enhancing phytochemicals (such as polyphenolic compounds) are also thought to be more abundant in GFB compared to grain-finished beef [10].

Differences in the nutritional profile between grass- and grain-finished beef have been studied extensively [6,7,8,11,12,13,14]. However, these studies mostly focused on a limited number of nutrients, such as fatty acids (FAs), minerals, and vitamins. Phytochemicals are secondary compounds produced by plants which may have health-improving attributes, such as antioxidant and anti-inflammatory effects [10,15]. Phenolics are secondary compounds derived from plants which contain at least one aryl ring bearing at least one hydroxyl group [16]. There are more than 8000 different phenolic compounds, and they can be classified as either non-flavonoids (phenolic acids), flavonoids, or tannins [15,17,18,19]. Although not crucial for primary biological processes, they still offer health benefits especially when they act as antioxidants and work as chain breakers or radical scavengers [18,20]. Polyphenolic compounds may protect cells against oxidative damage leading to the protection against various metabolic diseases caused by oxidative stress [21]. In the U.S., polyphenol intake reflects the low consumption of fruits and vegetables [22]. Based on these findings, it becomes crucial to identify foods and production practices that can be sources of polyphenols.

Ruminant products may contribute to overall dietary intake of polyphenols albeit at much lower concentrations than direct plant consumption, but also may provide secondary metabolites that are otherwise not commonly consumed in the human diet [10]. Earlier studies indicated that phenolic compounds present in the diet accumulate in the milk and meat of ruminants [10,15,16,23]. Polyphenols found in milk and meat mostly come from the plants animals forage on; the polyphenolic profile of milk and meat varies depending on the plant species present in the animal’s diet [10]. Numerous studies reported higher total phenolic content and antioxidant activity in milk and meat from ruminants foraging on pastures compared to concentrate or mixed diets [24,25,26,27]. Diverse pastures are usually higher in chlorophyll, carotenoids, and phenols than concentrate grain diets [28]. More specifically, individual plant species found in diverse pastures have different effects on bioactive compounds. In one study, alfalfa was positively correlated with phenolics, orchard grass was positively correlated with chlorophyll B but negatively correlated with carotenoids, and meadow fescue was negatively correlated with chlorophyll B [28]. Different kinds of pastures with differing plant species also display varying polyphenolic profiles [29]. These findings indicate that different plant species present in ruminant diets may result in varying transfer rates of bioactive compounds from plants to meat.

There has been interest in using plant byproducts and waste from the food industry to enhance the nutritional profile of meat [30,31,32,33]. In wine-producing regions, like Michigan, great quantities of waste and byproducts are generated causing economic and ecological issues [31]. Grape byproducts such as grapeseed extract (GSE) are rich in polyphenols, including anthocyanins, proanthocyanins, and flavanols, and can be fed to animals to increase the polyphenolic content and the oxidative stability of FAs in meat [30,31,34]. Feeding GSE directly to animals rather than incorporating it during meat processing ensures that bioactive compounds remain bioavailable and can be metabolized by the animal [35]. A previous study found a dose-dependent increase in muscle polyphenols when GSE was fed to rats [36]. Further, it was reported that the polyphenols found in grape byproducts were absorbed in sufficient amounts to modulate antioxidant activities in chicken muscles [31]. An additional study in chickens found that GSE polyphenols were absorbed and remained bioactive in chicken meat [30]. These findings indicate that feeding GSE to animals might result in meat with extended shelf-life, higher levels of PUFAs, and higher concentrations of polyphenols [36].

While there were attempts to identify and quantify polyphenolic compounds in goat milk and cheese [37,38], chicken meat [30,31], and cow milk [39], only a few studies focused on phytochemicals found in beef. A study conducted recently compared the secondary nutritional profiles of GFB and a plant-based alternative (including phenolic compounds) and found a 90% difference between both products, indicating that beef can accumulate compounds distinct from plant foods [23]. Additionally, another recent study reported higher levels of polyphenolic compounds in pasture-finished bison compared to grain-finished bison [40]. These studies confirmed that phytochemical compounds from the animal’s diet accumulate in their milk and meat, and that it is possible to identify and quantify these bioactive compounds. However, only a few studies focused on differences in phytochemicals between grass- and grain-finished beef. Therefore, the aim of the present study was to compare vitamins, minerals, FAs, and phytochemicals found in beef finished on a diverse pasture or on grain with or without GSE.

## 2. Materials and Methods

The animal use and procedures for this study have received the approval from the Institutional Animal Care and Use Committee at Michigan State University (IACUC #201800155).

### 2.1. Experimental Design, Animals, and Diets

This two-year study (2019 and 2020) was conducted at the Upper Peninsula Research and Extension Center (UPREC) (Michigan State University) located in Chatham, MI (latitude: 46°20′ N, longitude: 86°55′ W; elevation: 271 m). Three treatments were tested: a complex pasture mixture (GRASS), a total mixed feedlot ration (GRAIN), and a total mixed feedlot ration supplemented with 5% (dry matter—DM) GSE for the last 30 days of finishing (GRAPE). The experimental design for the GRASS and GRAIN groups was previously described [6,41]. Forty (*n* = 40) and thirty-two Red Angus steers (*n* = 32) (14–20 months of age) were randomly allocated to the diets in 2019 and 2020, respectively. Thirty steers in 2019 and twenty-two steers in 2020 were randomly stratified and allocated to one of three pens for each of the GRASS and GRAIN treatments (for each diet and each year, *n* = 5 animals per pen, with three pens). This design was followed in 2019, but due to lower male births in 2020, only two groups (one group with three steers and another group with four steers) were formed for the GRAIN diet. For GRAPE, ten Red Angus steers each year were kept in one same feedlot pen.

For this manuscript, for each year, nine samples per diet were randomly chosen. For GRASS and GRAIN in 2019, three samples per pen per year were randomly picked. The same design was followed for GRASS in 2020. Since only two pens for GRAIN were formed in 2020 due to lower male births, all seven samples were picked with an additional two random samples from 2019. Since GRAPE samples all came from the same pen, nine samples for each year were randomly chosen. The total number of beef samples analyzed for this study was 54 (*n* = 54).

The botanical composition of the diets was described previously [6,28]. Briefly, the plant species found in GRASS were meadow fescue (*Schedonorus pratensis* (Huds.) P. Beauv.), red clover (*Trifolium pratense* L.), timothy grass (*Phleum pratense*), alfalfa (*Medicago sativa*), white clover (*Trifolium repens* L.), birdsfoot trefoil (*Lotus corniculatus*), chicory (*Cichorium intybus*), orchardgrass (*Dactylis glomerata* L.), and dandelion (*Taraxacum oficinale* L.). The GRAIN diet was composed of orchardgrass hay, dry corn, high-moisture corn, and pellets (36% crude protein). The nutritional profiles of the GRASS and GRAIN diets were previously described by Krusinski et al. [28]. For GRAPE, 5% of GSE (DM basis) was added to GRAIN during the last 30 days of the finishing period. GSE was obtained from Pioneer Enterprises (Lewiston, ID, USA).

### 2.2. Sample Collection

Feed samples were collected every two weeks as described previously [28] (GRAPE samples were collected at the same frequency as GRAIN samples). For FA analysis, the number of feed samples was 21 GRASS, 15 GRAIN, and 7 GRAPE in 2019 and 24 GRASS, 10 GRAIN, and 5 GRAPE in 2020. Since these feed samples were also analyzed as part of other projects, fewer samples were left for phytochemical analysis: 11 GRAIN samples and 4 GRAPE samples were analyzed. Unfortunately, no GRASS samples were left for phytochemical analysis. GRASS samples underwent multiple runs as part of a series of previous experiments [28], leaving little to no samples for phytochemical analysis.

For beef sample collection, each year animals were harvested on the same day (late September in 2019 and early October in 2020) in a United States Department of Agriculture (USDA)-regulated facility (16–18 months old for GRAIN and GRAPE and 24–26 months old for GRASS), and ribeye samples were collected between the 11th and 13th rib on the left side of the carcass. Beef samples were then further processed at the Michigan State University Meat Laboratory; steaks were cut in 1 × 1 cm cubes and flash frozen with liquid nitrogen. Beef samples were stored at −80 °C until further analysis.

### 2.3. Proximate Analysis

Feed samples underwent proximate analysis based on protocols described by Maciel et al. [41] and Krusinski et al. [28]. Briefly, samples were dried in a forced-air oven at 105 °C for 8 h. Ash content was measured by oxidizing feed samples at 500 °C in a muffle furnace. Protocols by Mertens [42], which involved the addition of amylase and sodium sulfite, were followed to determine neutral detergent fiber (NDF). Acid detergent fiber (ADF) was analyzed following protocols described in AOAC [43]. Crude protein (CP) was measured as previously described [44]. Finally, gross energy was determined using a bomb calorimeter.

### 2.4. Fatty Acid Analysis

The protocol was described previously [6,28,32,45]. Unless otherwise specified, chemicals for FA analysis were obtained from Sigma-Aldrich (St. Louis, MO, USA). A microwave-assisted extraction was performed as detailed by Bronkema et al. [46]. Lyophilized and ground feed samples and minced beef samples were mixed with 8 mL 4:1 ethyl acetate:methanol (*v*/*v*) and underwent extraction in a CEM Mars 6 microwave digestion system equipped with a 24-vessel rotor and GlassChem tubes (CEM Corp., Matthews, NC, USA). Samples were then filtered using Whatman filter paper grade 597 into another set of test tubes containing 3.5 mL HPLC water and centrifuged to separate the top organic layer. The top layer was transferred to another set of tubes and evaporated under nitrogen gas. Samples were reconstituted with 4:1 dichloromethane:methanol (*v*/*v*) to reach a concentration of 20 mg of oil/mL.

A modified protocol from Jenkins [47] was used for the creation of FA methyl esters (FAMEs). Briefly, 100 μL containing 2 mg of oil was resuspended in toluene with 20 μg of internal standard (methyl 12-tridecenoate, U-35M, Nu-Chek Prep, Elysian, MN, USA). After resuspending, 2 mL of 0.5 N anhydrous potassium methoxide was added before being heated at 50 °C for 10 min. Then, 3 mL of methanolic HCl (5%) was added before heating the samples at 80 °C for 10 min. After cooling down, 2 mL of HPLC water and 2 mL of hexane were added, and samples were centrifuged at 2500 RPM for 5 min. The top layer was removed and dried under nitrogen gas. Next, FAMEs were resuspended in 1 mL of isooctane (final concentration was 2 mg/mL). Samples were then transferred to gas chromatography-mass spectrometry (GC-MS) vials with glass inserts.

For FAMEs quantification, a PerkinElmer (Waltham, MA, USA) 680/600S GC-MS in electron impact mode (70 eV) was used. The GC-MS was equipped with a HP-88 column (100 m, 0.25 mm ID, 0.2 μM film thickness) from Agilent Technologies (Santa Clara, CA, USA). One μL of feed samples was injected (GC temperature: 250 °C). One μL of beef samples was injected twice (20:1 split) (GC temperatures: 175 °C and 150 °C). For detailed temperature settings, please see Krusinski et al. [32]. A third injection in splitless mode followed for beef samples. This GC-MS method was modified from Kramer et al. [48]. The carrier gas was helium (1 mL/min), and MS data were recorded in full scan mode (m/z 70–400 amu).

FAMEs identification was achieved using MassLynx V4.1 SCN 714 (Water Corp., Milford, MA, USA). Retention time and EI mass fragmentation were compared to a reference standard containing Supelco 37 Component FAME Mix with mead acid, docosatetraenoic acid, *n*-3 DPA, *n*-6 DPA, and palmitelaidic acid (Cayman Chemical, Ann Arbor, MI, USA). The CLA standard UC-59M from Nu-Chek Prep (Elysian, MN, USA) was used for the identification of CLA isomers. For FAs not included in the standards, elution order and EI mass fragmentation were used for identification. FAMEs were quantified by utilizing a standard curve containing reference and internal standards.

### 2.5. Vitamin E and Mineral Analysis of Beef

The protocols for vitamin E and mineral analysis of beef were previously reported [32,46]. For vitamin E, the protocol was adapted from Rettenmaier and Schüep [49]. In short, 1 g of beef was thoroughly mixed with 5 mL of water. Proteins were precipitated with ethanol, and fat-soluble vitamins were extracted using hexane. Samples were centrifuged and the top layer was evaporated. Evaporated samples were resuspended in the chromatographic mobile phase and placed in vials. A six-point curve was made by serial diluting a vitamin E solution (Sigma-Aldrich, St. Louis, MO, USA) in ethanol (50 μg/mL to 0.2 μg/mL). A Waters Acquity system equipped with a Symmetry C18, 1.7 μm, 2.1 × 50 mm analytical column and Water Empower Pro Chromatography Manager software (Water Corp., Milford, MA, USA) were used for chromatography analysis. The mobile phase was acetonitrile:methylene chloride:methanol (70:20:10, *v*/*v*/*v*) and the flow rate was 0.5 mL/min. Detection was performed at UV absorption 292 nm.

The mineral analysis protocol was adapted from Wahlen et al. [50] and was previously described [6,32]. Briefly, the Agilent 7900 Inductivity Coupled Plasma-Mass Spectrometer (ICP-MS) (Agilent Technologies, Inc., Santa Clara, CA, USA) was used, and a six-point calibration curve and bovine liver and mussel standards (National Institute of Standards and Technology, Gaithersburg, MD, USA) were used as controls.

### 2.6. Polyphenolic Profiling

Feed samples underwent freeze-drying for 18.5 h using a Harvest Right Home Freeze Dryer (Harvest Right, North Salt Lake, UT, USA) and were subsequently ground through a 1 mm screen in a Wiley mill (Arthur H. Thomas, Philadelphia, PA, USA) with dry ice as reported by Krusinski et al. [28]. Beef samples were minced and pulverized on dry ice using a mortar and pestle. UHP-LC-MS-grade acetonitrile, methanol, DMSO, formic acid and water (Supelco LiChrosolv^®^) were ordered from Sigma-Aldrich (St. Louis, MO, USA). A QReSS™ internal standards kit, containing a mixture of isotopically labeled metabolites, was purchased from Cambridge Isotope Laboratories (Tewksbury, MA, USA). Purified external standards of compounds were obtained from Sigma-Aldrich (St. Louis, MO, USA) and/or Cayman Chemical (Ann Arbor, MI, USA).

The protocol by van Vliet et al. [40] was followed for this analysis. Briefly, 200 mg of pulverized beef and 50 mg of feed samples were mixed with 1000 μL and 500 μL, respectively, of methanol. At this time, 10 μL of QReSS™ internal standard (Cambridge Isotope Laboratories, Inc., Tewksbury, MA, USA) was added to the samples. Proteins were then precipitated under vigorous shaking for 10 min at 20 Hz using a QIAGEN TissueLyser II operated with two 5 mm glass beads (QIAGEN Sciences, Germantown, MD, USA). Samples were then centrifuged at 23,000× *g* for 10 min at 4 °C after a 1 h protein freeze-out step at −20 °C. Supernatants were removed and transferred to a new set of tubes. Beef samples were diluted with 2 mL of water with 1% formic acid (*v*/*v*), while feed samples were diluted with 1 mL of the same mixture. Strata C18-E cartridges (Phenomenex, Torrance, CA, USA) were used for solid phase extraction (SPE). Cartridges were activated with 1 mL of methanol with 1% formic acid and washed with 1 mL of water with 1% formic acid. Samples were then loaded onto the cartridges. After passing the samples through the cartridges, beef samples were washed with 2 mL of water with 1% formic acid while feed samples were washed with 1.2 mL of water with 1% formic acid. Beef and feed samples were eluted with 1.2 mL of methanol in 0.1% formic acid. Beef and feed samples were then evaporated under a gentle stream of nitrogen gas before being reconstituted with 100 μL and 200 μL, respectively, of methanol in 0.1% formic acid in 1.5 mL LC-MS amber vials (Agilent, Santa Clara, CA, USA) with 250 μL glass inserts.

Compounds were detected concurrently as precursor ion/product ion pair through multiple reaction monitoring (MRM) using ultraperformance liquid chromatography tandem mass spectrometry (UPLC-MS/MS). The platform utilized a SCIEX Hybrid Triple Quad™ 7500 (Framingham, MA, USA) with a front-end Shimadzu Nexera LC-40 Series (Kyoto, Japan) liquid chromatography system. Sample extracts were held at 10 °C in an auto-sampler, and compounds were separated at 30 °C using a reverse phase Kinetex F5 100Å column (2.1 mm × 150 mm, 2.6 μM) from Phenomenex (Torrance, CA, USA) with binary mobile phases of water (A) and acetonitrile (B), both containing 0.1% formic acid (*v*/*v*). Samples were run in both negative and positive electrospray ionization mode. The following source parameters were used in negative mode: 1600 V for the ionspray voltage, 550 °C for the temperature, 40 psi for the curtain gas, 40 psi for the nebulizer gas (GS1), 60 psi for the heating gas (GS2). In the negative mode, the linear gradient consisted of an initial composition of 5% B for 2.1 min with a flow rate of 0.2 mL/min, which was ramped up gradually to 95% B and a maximum flow rate of 0.46 mL/min over 14 min to keep a constant pressure, prior to being switched to 5% B for the final 4 min with a minimum flow rate of 0.175 mL/min.

The following source parameters were used in positive mode: 2000 V for the ionspray voltage, 550 °C for the temperature, 40 psi for the curtain gas, 40 psi for the nebulizer gas (GS1), 60 psi for the heating gas (GS2). In the positive mode, the linear gradient consisted of an initial composition of 5% B for 2.1 min with a flow rate of 0.2 mL/min, which was ramped up gradually to 95% B and a maximum flow rate of 0.46 mL/min over 14 min to keep a constant pressure, prior to being switched to 5% B for the final 4 min with a minimum flow rate of 0.175 mL/min. For both modes, a pooled matrix sample (sample generated by taking a small volume from samples from different experimental conditions), a double-blank (100% methanol), and mixture of purified standards of target compounds was injected using an unscheduled method to determine presence of compounds in the matrix sample and their retention times for the scheduled method. In both modes, the cycling time in the scheduled method was set to 1000 msec and the dwell time ranged from 3 to 250 msec depending on the number of MRMs triggered. Double-blank (100% methanol) and blank internal standard samples (methanol spiked with QReSS™ isotopically labeled internal standards) were run every 15 samples for quality control purposes.

Analyst 3.1 software (AB Sciex, Framingham, MA, USA) was used to acquire and analyze the chromatographic data. Peaks were integrated using area under the curve and normalization was performed using QReSS™ isotopically labeled internal standards to account for any loss of material during sample preparation. Unlabeled external standard mixes were run in parallel to the samples with known concentrations of the different metabolites to allow for quantitation (in mg/100 g) of various compounds with relevant nutritive value and for which a standard was available. For compounds with no relevant nutritive value or for which no standard was ran concurrently, the data is expressed as arbitrary units (AU).

### 2.7. Statistical Analysis

RStudio (R Core Team, Vienna, Austria) was used for the statistical analysis. A linear regression model was used to test the effect of diet on fatty acids, micronutrients, and quantified phytochemicals in beef. Diet, year, and pen were considered fixed effects. The experimental unit was each animal. Tukey’s HSD was used for post hoc comparison. For feed samples, a *t*-test was performed to compare GRAIN vs. GRAPE samples. For all analyses, values that fell below the limit of detection were treated as zeroes. Results were considered significant at *p* < 0.05 and were reported as mean ± standard error from the mean (SEM).

Principal component analysis (PCA) and random forest (RF) analysis were conducted using MetaboAnalyst 5.0 (metaboanalyst.ca) as described previously [23]. For these, phytochemical compounds were first normalized to mass and then log-transformed. The goal was to visualize data sets and identify the top metabolites that discriminate between groups using mean decrease accuracy.

## 3. Results

### 3.1. Nutritional Composition of the Diets

The proximate composition and the FA profile of the diets are displayed in Table 1. Overall, significant differences were seen between GRASS and the other two diets. GRAIN and GRAPE did not significantly differ. The two TMR diets (GRAIN and GRAPE) contained more DM than GRASS (*p* < 0.001). On the other hand, GRASS contained more ash, CP, NDF, ADF, and gross energy compared to the other two diets (*p* < 0.001). Regarding FAs, GRASS contained significantly more SFAs compared to GRAIN and GRAPE (*p* < 0.001). More specifically, differences were observed for C12:0 through C15:0 and C20:0 through C24:0 (*p* < 0.001 for all). GRAIN and GRAPE contained almost five times more MUFAs than GRASS (*p* < 0.001), even though GRASS displayed more palmitoleic acid (C16:1). GRAIN and GRAPE contained seven times more oleic acid than GRASS (*p* < 0.001). Significant differences were also observed for PUFAs with GRASS containing more total PUFAs than GRAIN and GRAPE (*p* < 0.001). More specifically, GRASS contained fifteen times more *n*-3 PUFAs compared to the other two diets (*p* < 0.001), while GRAIN and GRAPE contained higher levels of *n*-6 PUFAs compared to GRASS (*p* < 0.001). This was reflected in the *n*-6:*n*-3 ratio of the diets with GRAIN and GRAPE having a ratio 63 and 75 times higher than GRASS, respectively (*p* < 0.001). The phytochemical profiles of GRAIN and GRAPE feed samples are reported in Supplementary Table S1. Briefly, statistical differences were only observed for niacin and betaine, which were both higher in GRAIN compared to GRAPE.

### 3.2. Fatty Acid and Micronutrient Content of Beef

#### 3.2.1. Fatty Acids

The FA profile of beef by diet is displayed in Table 2. No significant differences were observed for total SFAs and individual SFAs (*p* > 0.05). Regarding MUFAs, no significant differences were reported for total MUFAs (*p* > 0.05), but beef from the GRAIN group contained more C14:1 9*c* than beef from GRASS, with beef from GRAPE not containing significantly different concentrations of this MUFA compared to the other two groups (*p* < 0.05). Additionally, differences were observed for C16:1 9*t* and C18:1 11*t* with beef from the GRASS group having higher concentrations of these FAs compared to beef from the other two groups (*p* < 0.001). Most differences were seen for *n*-3 and *n*-6 PUFAs. Beef from GRASS contained ~4.5 times and ~6 times more total *n*-3 PUFAs than beef from GRAIN and GRAPE, respectively (*p* < 0.001). Beef from GRASS contained higher concentrations of α-linolenic acid (ALA), EPA, and DPA than beef from the other two groups (*p* < 0.001). For DHA, significant differences were observed between beef from GRASS and beef from GRAIN but not for beef from GRAPE (*p* < 0.05). For total *n*-6 PUFAs (*p* < 0.05) and some individual *n*-6 FAs, beef from GRAIN contained more of this class of PUFAs than beef from GRASS, while beef from GRAPE did not significantly differ from the other two groups. These differences were reflected in the *n*-6:*n*-3 ratio with beef from GRASS having the lowest ratio (1.65:1) and beef from GRAIN (8.39:1) and GRAPE (9.82:1) having the highest (*p* < 0.001). Finally, no differences were observed for CLA, AD, and total FA (*p* > 0.05) between treatments.

#### 3.2.2. Micronutrients

**Figure 1:**
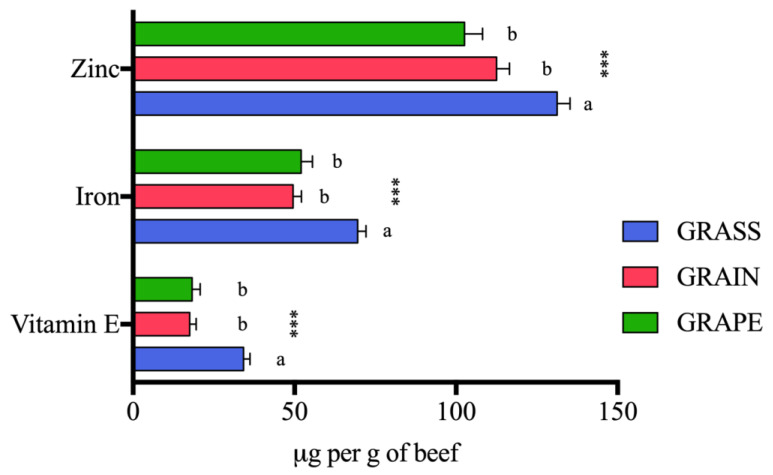
Main micronutrient concentrations in beef by diet (μg per g of beef). Values are reported as means ± standard error. Different letters denote statistical significance at *p* < 0.05 (‘***’ *p* < 0.001). GRASS: beef fed a diverse pasture; GRAIN: beef fed a total mixed ration (TMR); GRAPE: beef fed TMR + 5% DM grapeseed extract.

Main micronutrient concentrations in beef by diet are shown in Figure 1. Beef from GRASS contained more vitamin E, iron, and zinc compared to beef from the other two groups (*p* < 0.001 for all). No differences were observed between beef from GRAIN and beef from GRAPE (*p* > 0.05).

### 3.3. Phytochemical Profile of Beef

#### 3.3.1. Data Visualization and Identification of Top Discriminating Compounds

**Figure 2:**
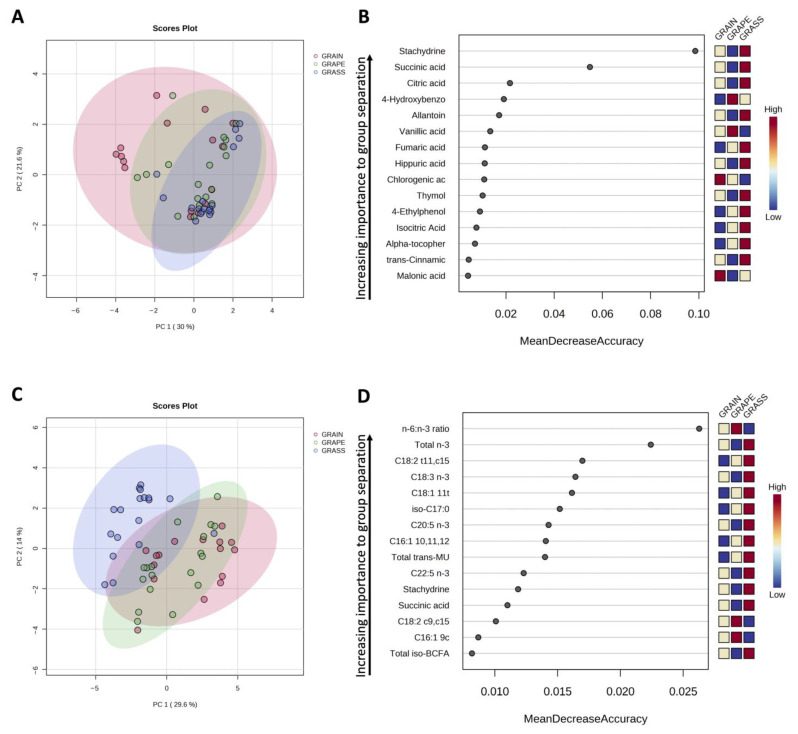
(**A**) Principal component analysis (PCA) plot using phytochemicals only showing separation and clusters based on finishing diet with some overlaps. (**B**) Random forest (RF) variable importance plot showing main phytochemicals capable of discriminating beef based on finishing diet. (**C**) PCA plot using phytochemicals and fatty acids showing three clusters based on finishing diets. (**D**) RF variable importance plot showing main fatty acids and phytochemicals capable of discriminating beef based on finishing diet. For RF plots, the y-axis represents phytochemicals in order of importance for group classification (from top to bottom). The x-axis shows mean decrease accuracy, with a higher value indicating the importance of that phytochemical in predicting groups. PC: principal component; total *n*-3: total omega-3 fatty acids; total *trans*-MU: total *trans* monounsaturated fatty acids; total *iso*-BCFA: total *iso* branched chain fatty acids; GRASS: beef fed a diverse pasture; GRAIN: beef fed a total mixed ration (TMR); GRAPE: beef fed TMR + 5% DM grapeseed extract.

Results from the PCA and RF analysis are displayed in Figure 2. PCA (A) displayed separation between beef from GRASS, GRAIN, and GRAPE as shown by the 30% variation along principal component 1. Three clusters corresponding to the three finishing diets can be observed on the PCA plot, with overlaps. The RF plot (B) highlighted the results from the PCA, showing distinctions in specific phytochemicals in beef by diets. Stachydrine and succinic acid were the two most discriminating phytochemicals, followed by citric acid, 4-hydrobenzoic acid, allantoin, and vanillic acid. RF classification also showed good prediction of group with an overall out-of-bag (OOB) error rate of 17.30% (Table 3). PCA (C) showed separation between beef coming from the three different finishing systems, highlighted by clusters and 29.60% variation along principal component 1. When FAs were included in the RF analysis, the *n*-6:*n*-3 ratio and total *n*-3 PUFAs were the two most discriminating factors. Stachydrine and succinic acid still remained in the top 15 most important compounds to separate beef by diet. The OOB was also reduced from 17.30% to 15.40% when FAs were included in the model (Table 3).

#### 3.3.2. Differences in Quantified Phytochemicals in Beef

Quantified phytochemicals in beef by diet (in mg per 100 g of beef) are displayed in Table 4. Beef from GRASS contained higher levels of stachydrine, hippuric acid, citric acid, succinic acid, and fumaric acid compared to beef from GRAIN and GRAPE (*p* < 0.001 for all). These results complete the findings from the RF analysis reported above. Beef from GRAIN and GRAPE contained higher concentrations of p-coumaric acid than beef from GRASS (*p* < 0.05). No differences by diet were observed for the rest of the quantified phytochemicals (*p* > 0.05).

## 4. Discussion

### 4.1. Nutritional Composition of the Diets

Differences in nutritional composition between pasture and TMR were reported by Krusinski et al. [28]. Grasses usually contain higher levels of SFAs and PUFAs (especially *n*-3) when compared to grains [51,52]. Higher concentrations of *n*-3 PUFAs in grasses are due to the accumulation of such FAs in leaf tissue of fresh pasture, with levels depending on the leaf-to-stem ratio [53,54,55]. Forages usually contain 50–75% of *n*-3 PUFAs as part of their FA composition [56]. Findings in the present study align with these numbers, with GRASS containing ~61% of *n*-3 PUFAs. Grains are usually higher in MUFAs and *n*-6 PUFAs when compared to grasses. This is mainly due to the growth of grain ears and the accumulation of these FAs in those ears [53]. In the present study, more than 50% of FAs in GRAIN and GRAPE were *n*-6 PUFAs. The concentrations of *n*-3 and *n*-6 PUFAs in the diets were ultimately reflected in the *n*-6:*n*-3 ratio which was significantly lower in GRASS compared to the other two TMR diets. While such differences were anticipated between grasses and TMR, more differences were expected between GRAIN and GRAPE as grapeseed oil is composed of ~75% *n*-6 PUFAs [57]. However, since only 5% (DM basis) were added to the TMR for the GRAPE diet, it is possible that such amounts were too low to reflect a difference in the nutritional profile of the diets.

Vinyard et al. [58] included either 15% or 30% (DM basis) of grape pomace to a TMR diet and found that ADF and NDF increased with the concentration of grape pomace in the diet compared to TMR alone. However, Nudda et al. [59] reported similar proximate composition values between TMR and TMR with grape pomace, aligning with results presented in the current study. Even though not statistically significant, higher levels of (-) epicatechin gallate and epicatechin were observed in the GRAPE diet. This was expected, as grape byproducts usually provide polyphenolic compounds such as catechin, epicatechin, and procyanidins [60]. The lack of statistical significance may be attributed to the small sample size (*n* = 4) for GRAPE leading to large SEM.

### 4.2. Beef Fatty Acids and Micronutrients

#### 4.2.1. Fatty Acids

Differences in the FA profile of beef from grass and grain finishing systems were widely reported in the literature [6,8,12,61,62]. The absence of significant differences between groups regarding SFAs aligns with what others described [6,61]. While some reported that concentrations of SFAs in GFB are higher than grain-finished beef, this is mainly because FAs were reported as percent of total FAs [7,63]. GFB is generally leaner, resulting in no significant differences compared to concentrations of SFAs in grain-finished beef when reported as mg per 100 g of beef [17]. Manso et al. [64] reported a decrease in some SFAs in the milk of ewes supplemented with 10% (DM basis) of grape pomace compared to the milk of ewes fed a simple TMR/forage concentrate diet. However, differences were not observed when ewes were supplemented with only 5% (DM basis) of grape pomace, indicating a dose-dependent response. Moate et al. [65] reported similar findings in dairy cows and attributed this decrease to the presence of grape residues containing lignin which are not fermentable in the rumen. Since no decrease in SFAs was observed in the current study, it was most likely due to the lower dose of GSE added to the diet.

The lack of differences in total MUFA concentrations was unexpected since grain-finished beef generally contains 30–70% more MUFAs than GFB [7,17,61]. Krusinski et al. [6] reported similar results regarding individual MUFAs with GFB having higher levels of specific *trans*-MUFAs and grain-finished beef having higher concentrations of specific *cis*-MUFAs. When high levels of *trans*-MUFAs are reported in GFB, it is generally due to higher concentrations of beneficial vaccenic acid [6,52,66]. In general, MUFAs are of interest for their low-density lipoprotein (LDL) cholesterol-lowering potential [67] and for their contribution to the overall palatability of beef [68,69].

As expected, beef from GRASS contained more *n*-3 PUFAs (including ALA, EPA, and DPA) than beef from GRAIN and GRAPE. These long-chain PUFAs are associated with healthier cardiovascular and cognitive functions [70,71]. On the other hand, beef from GRAIN contained more *n*-6 PUFAs than beef from GRASS. This class of PUFAs may be pro-inflammatory compared to their *n*-3 counterpart, which may be anti-inflammatory [72]. Surprisingly, beef content of *n*-6 PUFAs was not different between GRAPE and GRASS nor GRAPE and GRAIN. Ianni et al. [73] noted that the inclusion of grape pomace in the diet of cattle usually results in higher proportions of LA in beef, mainly because grape byproducts contain great concentrations of this *n*-6 FA. However, Manso et al. [64] noted that the increase in LA in milk from ewes fed grape byproducts is dose-dependent and significant changes are seen when at least 10% (DM basis) of grape supplementation is added to the diet. The *n*-6:*n*-3 ratio is generally used for nutritional claims associated with GFB [13,17]. An ideal ratio for human health is hypothesized to be around 1:1–4:1 [72,74]. Higher *n*-6:*n*-3 ratios were associated with impaired growth and development [75], as well as obesity and weight gain in both human and animal studies [76]. Simopoulos [76] highlighted that a balanced *n*-6:*n*-3 ratio (1-2:1) may be one of the most important dietary factors to prevent obesity. Additionally, higher *n*-3 PUFA intakes are related to better cognitive development [75]. In this study, beef from GRASS had a more optimal *n*-6:*n*-3 ratio for human health (1.65:1) compared to the other two groups that had a ratio closer to 10:1 (a value sometimes associated with adverse health effects [74]). Some argued that using the *n*-6:*n*-3 ratio as health indicator is far too simplistic, and a proposed replacement is the “Omega-3 Index” which focuses mostly on EPA and DHA [77]. The Food and Agriculture Organization (FAO) also estimated that there is “no rationale for a specific recommendation for the *n*-6:*n*-3 ratio” as long as intakes of *n*-6 and *n*-3 PUFAs are sufficient [78]. According to the FAO, the intake of total *n*-3 PUFAs can range between 0.5 and 2% of energy (with the recommendation for EPA + DHA set at 2 g/day), and the intake of *n*-6 PUFAs can range between 2.5 and 9% of energy [78].

#### 4.2.2. Vitamin E, Zinc, and Iron

Higher levels of vitamin E, iron, and zinc are expected for GFB compared to grain-finished beef [6,79]. Higher concentrations of vitamin E in GFB are generally enough to protect meat from oxidation, leading to extended shelf-life [80]. The antioxidant potential of vitamin E also protects cells against free radicals, which can benefit human health [13,79]. Untea et al. [81] showed the oxidative stability-influencing parameters of grape pomace and noted that it contains significant amounts of vitamin E and zinc. Vitamin E is a free radical scavenger and breaks the chain of lipid peroxidation, but zinc can also protect cells from iron-initiated lipid oxidation [81]. It was expected that the addition of GSE to the cattle diet would increase zinc and vitamin E concentrations compared to TMR alone. However, no such differences were noted in the present study. There is most likely a dose-dependent effects for these compounds and the levels of GSE added were probably too low to observe significant differences.

### 4.3. Phytochemical Profile of Beef

**Figure 3:**
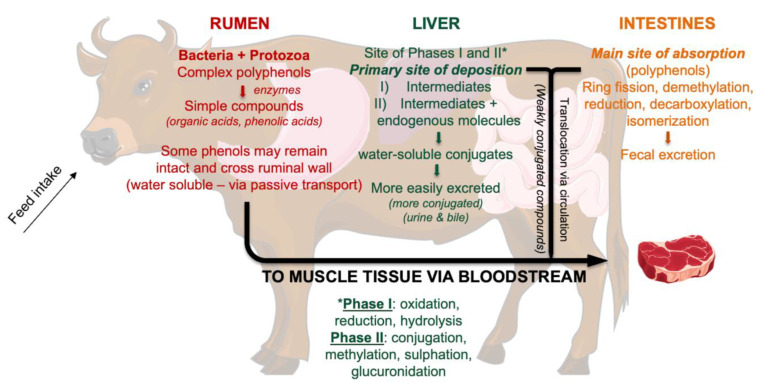
Phenolic compounds metabolism in ruminants. Information collected from [84,93,97,98,99]. It is generally assumed that the bioavailability of polyphenols coming from the small intestine is low (5–10% in monogastric animals [98]), because these compounds are treated as xenobiotics and undergo biotransformation in the body. Some phenols are absorbed in the rumen [100]. It is also important to note that a proportion of polyphenols may also come from amino acid catabolism in ruminants [84].

Beef samples tested in this study all came from similar genetics steers, indicating that differences observed were most likely due to differences in finishing diets (GRASS vs. GRAIN vs. GRAPE). One limitation from the current study is that the phytochemical profile of GRASS feed samples was not reported, so the extent of transfer of phytochemicals from plants to the meat cannot be established with certainty. O’Connell and Fox [16] stated that most polyphenolic compounds found in dairy products are derived from feeds, even though some of them may be the products of amino acid catabolism. While metabolism of such compounds in ruminants is not yet well understood, an illustration of the current knowledge is displayed in Figure 3.

Grasses are generally high in antioxidants, including vitamin E, chlorophyll, carotenoids, and phenols [28]. In the present study, beef from GRASS contained higher levels of numerous phytochemicals including stachydrine, hippuric acid, citric acid, and succinic acid compared to beef from GRAIN and GRAPE. These specific phytochemicals were also identified in the RF analysis as compounds capable of predicting diets. Stachydrine and hippuric acid were also identified as cattle-diet-discriminating compounds by others [40,82], even though van Vliet et al. [40] reported higher levels of stachydrine in pen-finished bison compared to pasture-finished bison, which can be explained by the high level of alfalfa in the finishing ration of pen-finished bison. This phytochemical is found in high concentrations in chestnuts, alfalfa, and Chinese medicinal herbs and demonstrates bioactivities that have potential applications in addressing fibrosis, cardiovascular diseases, cancers, brain diseases, and inflammation in humans [83]. Since considerable levels of stachydrine are found in alfalfa, it was expected to find higher concentrations of this compound in beef from GRASS since ~10% of the complex diverse pasture fed to these animals was made of alfalfa [6,28]. Besle et al. [39] identified hippuric acid as a major compound capable of indicating cattle finishing diets (with higher levels found in the milk from animals kept on grasslands). Higher concentrations of this phytochemical in the milk and meat of grass-finished animals may likely be a result of the presence of phenolic acids in their pasture-based diets [84]. Citric acid was the most abundant phytochemical quantified in this study (with beef from GRASS containing 379.20 mg of citric acid per 100 g of beef). Citric acid is mostly found in fruits, especially citrus fruits, and has several health benefits, including increasing the bioavailability and absorption of minerals and reducing risks of kidney stone formation [85,86]. Supplee and Bellis [87] noted that pasture feeding may increase concentrations of citric acid in milk in some instances. For comparison, fresh apricots contain 30–50 mg of citric acid per 100 g [88]. In the present study, 100 g of beef from GRASS contained 7–12 times more citric acid than 100 g of apricots. Succinic acid was also abundant in beef from GRASS. Gatmaitan et al. [89] reported a decrease in relative abundance of succinic acid in grain-finished beef and indicated that this compound can be used for the authentication of GFB. Succinic and citric acid are both TCA cycle metabolites that can also be endogenously produced. These are usually elevated with an oxidative phenotype in GFB due to movement and more long-chain PUFAs in the forage. Additionally, long-chain PUFAs are preferentially oxidized in the mitochondria. So, while feed plays a role in the current study, it is also likely that these compounds were endogenously produced because of cattle moving more and/or eating more PUFAs [90,91]. Beef from GRAIN and GRAPE (fed mainly a TMR) contained higher concentrations of p-coumaric acid than beef from GRASS. This phenolic acid is one of the main phenolic compounds reported in corn-based diets [84].

The PCA plot showed overlaps between clusters with the GRAPE group overlapping with GRAIN and GRASS, which may indicate a transfer of phytochemicals from the diet to the meat. The quantified phytochemicals presented in this study are not exclusive to GSE, which may explain why no significant differences were observed between beef from GRAPE and beef from the other two diets. Based on the RF biochemical importance plot, it appears that vanillic acid and 4-hydrobenzoic acid have the potential to discriminate beef from cattle supplemented with GSE even though no significant differences were noted when these compounds were quantified. Vanillic acid is one of the most significant hydrobenzoic acids found in grapes [59,92]. Whether supplementing cattle diets with GSE increases phytochemicals in beef remains uncertain, even though higher plasma polyphenols have been reported in cattle supplemented with grape byproducts [93]. Another important point is that the 5% (DM basis) of GSE added to the TMR may not be enough to observe significant changes. It appears that the effects of GSE on the beef nutritional profile are dose-dependent [36,64].

Overall, the differences in phytochemicals between grass- and grain-finished beef noted in this study agreed with what was previously reported on products from grazing animals compared to animals fed a conventional grain diet [10,37,39,40,82,94]. It is important to note that when FAs were included in the PCA, the plot showed more separation and clustering than with phytochemicals alone. Additionally, the RF analysis including FAs and phytochemicals identified the *n*-6:*n*-3 ratio as the most important factor to separate beef by finishing diet. Monahan et al. [95] and Prache et al. [96] also identified the *n*-6:*n*-3 ratio as an important marker of identification for GFB.

## 5. Conclusions

Overall, beef from the grass-finishing system (GRASS) displayed the most beneficial nutritional profile for human health with a lower *n*-6:*n*-3 ratio and higher levels of long-chain *n*-3 PUFAs, vitamin E, zinc, iron, and various secondary metabolites, including stachydrine, hippuric acid, citric acid, succinic acid, and fumaric acid compared to grain-finished beef (GRAIN) and grain-finished beef supplemented with GSE (GRAPE). Interestingly, beef from GRAPE was somewhere in the middle between GRASS and GRAIN for a few specific FAs such as total *n*-6 PUFAs, C20:3 *n*-3, and DHA. The same observation was made with PCA, where some overlaps were observed. Random forest classification allowed us to identify the most important phytochemicals for group separation, with stachydrine, succinic acid, and citric acid being the top three. When FAs were included, the *n*-6:*n*-3 ratio and total *n*-3 PUFAs were the two most important factors capable of group separation.

While higher levels of phytochemicals were expected with GSE supplementation, the response is generally dose-dependent and the 5% (DM basis) added to the cattle diet in this study were most likely not enough to raise levels of bioactive compounds in beef. The main limitation of this study was the limited profiling of phytochemicals of the diets, making the assumption of transfer of phytochemicals from plants to meat more difficult. We also did not quantify phytochemicals specific to grapes, which does not allow us to determine the deposition rate of such compounds in beef supplemented with GSE. However, this study displayed extensive nutritional profiles of beef from two common finishing systems (GRASS and GRAIN) and a third finishing diet worth exploring in more detail using a byproduct from the wine industry (GRAPE). We were also able to quantify phytochemicals and report them in amounts relevant for human health (mg per 100 g of beef). Future studies should investigate the addition of phytochemical-rich byproducts to cattle diets (and in varying amounts) and their effects on the nutritional profile of beef. Additionally, quantifying these bioactive compounds and reporting results that can be used by consumers is crucial. More research is needed to link livestock production systems, nutritional profiles of animal products, and human health. Such findings could also be used for the authentication of GFB.

In conclusion, the *n*-6:*n*-3 ratio, total *n*-3 PUFAs, micronutrients, and phytochemicals in beef are compounds that can be used to determine the finishing diet of cattle. GFB with higher amounts of beneficial bioactive compounds may favor human health.

## Figures and Tables

**Table 1:** Nutritional composition of the diets.

	GRASS ^1^	GRAIN ^2^	GRAPE ^3^	*p-V*alue
C10:0	0.03 ± 0.02	0.00 ± 0.00	0.00 ± 0.00	0.216
C12:0	0.21 ± 0.02 ^a^	0.03 ± 0.00 ^b^	0.03 ± 0.01 ^b^	<0.001
C14:0	0.66 ± 0.05 ^a^	0.07 ± 0.00 ^b^	0.07 ± 0.00 ^b^	<0.001
C15:0	0.13 ± 0.01 ^a^	0.03 ± 0.00 ^b^	0.03 ± 0.00 ^b^	<0.001
C16:0	14.13 ± 0.22	13.82 ± 0.20	13.35 ± 0.17	0.156
C16:1 *c*9	0.18 ± 0.02 ^a^	0.09 ± 0.01 ^b^	0.09 ± 0.01 ^b^	<0.010
C16:1 *c*7	1.06 ± 0.04 ^a^	0.14 ± 0.01 ^b^	0.12 ± 0.02 ^b^	<0.001
C17:0	0.20 ± 0.01 ^a^	0.06 ± 0.00 ^b^	0.07 ± 0.00 ^b^	<0.001
C18:0	1.73 ± 0.07	1.71 ± 0.05	1.67 ± 0.05	0.898
C18:1 *c*9	3.17 ± 0.26 ^b^	22.27 ± 0.14 ^a^	22.15 ± 0.31 ^a^	<0.001
C18:1 *c*11	0.52 ± 0.05	0.52 ± 0.01	0.48 ± 0.01	0.896
C18:2 *n*-6 (LA) ^4^	14.09 ± 0.42 ^b^	56.11 ± 0.57 ^a^	57.03 ± 0.64 ^a^	<0.001
C18:3 *n*-3 (ALA) ^5^	61.60 ± 0.93 ^a^	4.29 ± 0.30 ^b^	3.95 ± 0.66 ^b^	<0.001
C20:0	0.62 ± 0.03 ^a^	0.42 ± 0.02 ^b^	0.41 ± 0.04 ^b^	<0.001
C22:0	0.75 ± 0.04 ^a^	0.17 ± 0.01 ^b^	0.17 ± 0.01 ^b^	<0.001
C24:0	0.74 ± 0.03 ^a^	0.23 ± 0.01 ^b^	0.22 ± 0.01 ^b^	<0.001
∑ SFA ^6^	19.36 ± 0.38 ^a^	16.57 ± 0.27 ^b^	16.03 ± 0.24 ^b^	<0.001
∑ OCFA ^7^	0.33 ± 0.01 ^a^	0.09 ± 0.00 ^b^	0.10 ± 0.00 ^b^	<0.001
∑ MUFA ^8^	4.94 ± 0.25 ^b^	23.02 ± 0.13 ^a^	22.98 ± 0.30 ^a^	<0.001
∑ PUFA ^9^	75.71 ± 0.57 ^a^	60.41 ± 0.32 ^b^	60.99 ± 0.34 ^b^	<0.001
∑ *n*-6 ^10^	14.09 ± 0.42 ^b^	56.12 ± 0.57 ^a^	57.04 ± 0.63 ^a^	<0.001
∑ *n*-3 ^11^	61.62 ± 0.93 ^a^	4.29 ± 0.30 ^b^	3.95 ± 0.66 ^b^	<0.001
*n-*6:*n*-3 ratio ^12^	0.24 ± 0.01 ^b^	15.12 ± 1.28 ^a^	18.42 ± 2.34 ^a^	<0.001
DM ^13^	21.38 ± 0.63 ^b^	79.80 ± 0.97 ^a^	82.36 ± 1.32 ^a^	<0.001
Ash *	6.60 ± 0.17 ^a^	4.02 ± 0.20 ^b^	3.79 ± 0.34 ^b^	<0.001
CP ^14^*	13.40 ± 0.47 ^a^	9.65 ± 0.18 ^b^	9.29 ± 0.32 ^b^	<0.001
NDF ^15^*	51.82 ± 0.92 ^a^	21.02 ± 0.39 ^b^	20.59 ± 0.62 ^b^	<0.001
ADF ^16^*	33.38 ± 0.72 ^a^	10.09 ± 0.26 ^b^	9.94 ± 0.39 ^b^	<0.001
Energy ^17^	4465.49 ± 11.98 ^a^	4265.90 ± 15.98 ^b^	4274.76 ± 28.55 ^b^	<0.001

Values reported as means ± standard error. Different letters denote statistical significance at *p* < 0.05. ^1^ GRASS: diverse pasture; ^2^ GRAIN: total mixed ration (TMR); ^3^ GRAPE: TMR + 5% DM grapeseed extract. Fatty acids reported as % of total. ^4^ LA: linoleic acid; ^5^ ALA: α-linolenic acid; ^6^ ∑ SFA = all saturated FAs (10:0, 12:0, 13:0, 14:0, 15:0, 16:0, 17:0, 18:0, 20:0, 22:0, 24:0); ^7^ ∑ OCFA = all odd chain FAs (13:0, 15:0, 17:0); ^8^ MUFA = all monounsaturated FAs (16:1, 18:1); ^9^∑ PUFA = LA + ALA + C20:3 *n*-3; ^10^ ∑ *n*-6 = LA; ^11^∑ *n*-3 = ALA + C20:3 *n*-3; ^12^ *n*-6:*n*-3 ratio = ∑ *n*-6/∑ *n*-3; * reported in %DM; ^13^ DM: dry matter (%); ^14^ CP: crude protein; ^15^ NDF: neutral detergent fiber; ^16^ ADF: acid detergent fiber; ^17^ Energy (cal/g).

**Table 2:** Fatty acid profile of beef by diet (mg per 100 g beef).

	GRASS ^1^	GRAIN ^2^	GRAPE ^3^	*p*-Value
∑ SFA ^4^	767.56 ± 188.89	996.16 ± 194.19	868.22 ± 266.53	0.700
C12:0	1.04 ± 0.25	1.09 ± 0.26	0.63 ± 0.35	0.527
C14:0	31.29 ± 10.15	53.81 ± 10.43	49.95 ± 14.32	0.287
C15:0	8.16 ± 2.23	3.97 ± 2.29	3.34 ± 3.15	0.330
C16:0	424.06 ± 107.99	681.64 ± 111.01	564.21 ± 152.37	0.261
C17:0	26.17 ± 8.62	10.18 ± 8.86	23.16 ± 12.16	0.403
C18:0	272.22 ± 66.47	242.23 ± 68.33	224.09 ± 93.79	0.907
∑ MUFA ^5^	748.55 ± 194.06	944.75 ± 188.04	1076.92 ± 258.41	0.572
C14:1 9*c*	5.57 ± 3.30 ^b^	19.10 ± 3.39 ^a^	13.67 ± 4.65 ^a,b^	<0.050
C16:1 9*c*	60.47 ± 54.83	117.74 ± 56.37	240.76 ± 77.37	0.174
C16:1 9*t*	3.51 ± 0.33 ^a^	1.08 ± 0.20 ^b^	1.05 ± 0.27 ^b^	<0.001
C18:1 9*c*	565.28 ± 148.34	733.76 ± 152.50	732.59 ± 209.30	0.694
C18:1 9*t*	2.24 ± 0.95	1.44 ± 0.27	1.75 ± 0.36	0.578
C18:1 11*t*	36.69 ± 11.11 ^a^	1.54 ± 2.02 ^b^	1.70 ± 2.70 ^b^	<0.001
∑ PUFA ^6^	113.84 ± 12.60	111.65 ± 12.95	95.29 ± 17.78	0.669
∑ *n*-3 ^7^	46.03 ± 4.79 ^a^	10.13 ± 4.92 ^b^	7.73 ± 6.76 ^b^	<0.001
C18:3 *n*-3 (ALA) ^8^	24.32 ± 3.70 ^a^	2.32 ± 3.81 ^b^	2.22 ± 5.23 ^b^	<0.001
C20:3 *n*-3	0.30 ± 0.07 ^a^	0.03 ± 0.07 ^b^	0.09 ± 0.09 ^a,b^	<0.050
C20:5 *n*-3 (EPA) ^9^	7.41 ± 0.38 ^a^	1.80 ± 0.39 ^b^	1.10 ± 0.53 ^b^	<0.001
C22:5 *n*-3 (DPA) ^10^	13.39 ± 0.96 ^a^	5.67 ± 0.99 ^b^	3.96 ± 1.36 ^b^	<0.001
C22:6 *n*-3 (DHA) ^11^	0.61 ± 0.08 ^a^	0.32 ± 0.08 ^b^	0.35 ± 0.11 ^a,b^	<0.050
∑ *n*-6 ^12^	67.07 ± 9.04 ^b^	100.32 ± 9.29 ^a^	86.61 ± 12.73 ^a,b^	<0.050
C18:2 *n*-6 (LA) ^13^	46.95 ± 7.07	65.61 ± 7.27	57.47 ± 9.97	0.195
C18:3 *n*-6	0.29 ± 0.05	0.40 ± 0.05	0.42 ± 0.07	0.243
C20:2 *n*-6	0.33 ± 0.09	0.57 ± 0.10	0.55 ± 0.13	0.186
C20:3 *n*-6	3.34 ± 0.49 ^b^	5.87 ± 0.50 ^a^	4.61 ± 0.69 ^a,b^	<0.010
C20:4 *n*-6	14.61 ± 1.51 ^b^	22.53 ± 1.56 ^a^	19.21 ± 2.14 ^a,b^	<0.010
C22:4 *n*-6	1.56 ± 0.49 ^b^	5.34 ± 0.51 ^a^	4.36 ± 0.70 ^a^	<0.001
*n*-6:*n*-3 ratio ^14^	1.65 ± 0.44 ^b^	8.39 ± 0.45 ^a^	9.82 ± 0.62 ^a^	<0.001
∑ CLA ^15^	5.14 ± 1.20	2.28 ± 1.23	2.86 ± 1.69	0.243
C18:2 9*c*,11*t*/9*c*,7*t*	4.01 ± 1.05	1.63 ± 1.08	1.96 ± 1.48	0.269
∑ AD ^16^	18.07 ± 5.07	9.29 ± 5.21	17.01 ± 7.16	0.438
∑ CLnA ^17^	0.36 ± 0.05	0.25 ± 0.05	0.25 ± 0.07	0.292
C18:3 9*c*,11*t*,15*t*	0.12 ± 0.03 ^a^	0.02 ± 0.03 ^b^	0.05 ± 0.04 ^a,b^	<0.050
C18:3 9*c*,11*t*,15*c*	0.24 ± 0.03	0.23 ± 0.03	0.20 ± 0.04	0.758
∑ FA ^18^	1686.08 ± 400.01	2080.23 ± 399.93	2090.96 ± 549.44	0.495

Values reported as means ± standard error. Different letters denote statistical significance at *p* < 0.05. ^1^ GRASS: beef fed a diverse pasture; ^2^ GRAIN: beef fed a total mixed ration (TMR); ^3^ GRAPE: beef fed TMR + 5% DM grapeseed extract; ^4^ ∑ SFA = all saturated FAs (10:0, 12:0, 13:0, 14:0, 15:0, 16:0, 17:0, 18:0, 19:0, 20:0, 22:0); ^5^ ∑ MUFA = all monounsaturated FAs (14:1, 16:1, 17:1, 18:1, 20:1); ^6^ ∑ PUFA = LA + ALA + GLA + Eicosadienoic + Eicosatrienoic + DGLA + Mead + Arachidonic + EPA + DTA + DPA *n*-3 + DHA; ^7^ ∑ *n*-3 = ALA + EPA + DHA + DPA *n*-3 + Eicosatrienoic; ^8^ ALA: α-linolenic acid; ^9^ EPA: eicosapentaenoic acid; ^10^ DPA: docosapentaenoic acid; ^11^ DHA: docosahexaenoic acid; ^12^ ∑ *n*-6 = LA + GLA + Eicosadienoic + DGLA + Arachidonic + DTA; ^13^ LA: linoleic acid; ^14^ *n*-6:*n*-3 ratio = ∑ *n*-6/∑ *n*-3; ^15^ ∑ CLA = sum of conjugated linoleic acid isomers (*c*9, *t*11/*t*7, *c*9 18:2 + *t*11, *c*13 18:2 + *t*11, *t*13 18:2 + *t*,*t* 18:2); ^16^ ∑ AD (Atypical Dienes) = sum of non-conjugated linoleic acid isomers (*t*11, *t*15 18:2 + *t*9, *t*12 18:2 + *c*9, *t*14/*c*9, *t*13 18:2 + *t*11, *c*15 18:2 + *c*9, *t*16 18:2 + *c*9, *c*15 18:2 + *c*12, *c*15 18:2); ^17^ ∑ CLnA = sum of conjugated linolenic acid isomers (*c*9, *t*11, *t*15 18:3 + *c*9, *t*11, *c*15 18:3); ^18^ ∑ FA = sum all of FAs.

**Table 3:** Random forest classification of beef by diet.

	GRASS ^1^	GRAIN ^2^	GRAPE ^3^	Class Error	OOB ^4^
Phytochemicals					
GRASS	17	1	0	5.60%	17.30%
GRAIN	0	10	6	37.50%	
GRAPE	0	2	16	11.10%	
Phytochemicals and fatty acids					
GRASS	17	1	0	5.60%	15.40%
GRAIN	0	13	3	18.80%	
GRAPE	1	3	14	22.00%	

^1^ GRASS: beef fed a diverse pasture; ^2^ GRAIN: beef fed a total mixed ration (TMR); ^3^ GRAPE: beef fed TMR + 5% DM grapeseed extract; ^4^ OOB: overall out-of-bag error rate.

**Table 4:** Quantified phytochemicals in beef by diet (mg per 100 g beef).

	GRASS ^1^	GRAIN ^2^	GRAPE ^3^	*p*-Value
Stachydrine	0.65 ± 0.04 ^a^	0.26 ± 0.05 ^b^	0.28 ± 0.06 ^b^	<0.001
4-Ethylphenol	3.92 ± 0.44	3.00 ± 0.45	3.83 ± 0.60	0.287
Hippuric acid	13.37 ± 1.20 ^a^	7.77 ± 1.24 ^b^	5.52 ± 1.66 ^b^	<0.001
Citric acid	379.20 ± 35.20 ^a^	79.10 ± 36.30 ^b^	51.40 ± 48.70 ^b^	<0.001
Succinic acid	36.80 ± 2.66 ^a^	17.80 ± 2.74 ^b^	13.70 ± 3.67 ^b^	<0.001
Fumaric acid	2.68 ± 0.34 ^a^	0.90 ± 0.35 ^b^	0.62 ± 0.47 ^b^	<0.001
Caffeic acid	0.04 ± 0.02	0.04 ± 0.02	0.01 ± 0.03	0.570
p-Coumaric acid	1.56 ± 0.25 ^b^	2.47 ± 0.25 ^a^	2.53 ± 0.34 ^a^	<0.050
4-Hydroxybenzoic acid	0.38 ± 0.22	0.02 ± 0.23	0.85 ± 0.30	0.058
Gallic acid	0.01 ± 0.02	0.00 ± 0.02	0.05 ± 0.03	0.370
Ethyl gallate	0.01 ± 0.01	0.01 ± 0.01	0.02 ± 0.01	0.871
Vanillic acid	0.18 ± 0.16	0.28 ± 0.16	0.38 ± 0.22	0.733
D-Tartaric acid	2.29 ± 0.52	2.34 ± 0.53	1.71 ± 0.71	0.734
Pyrocatechol sulfate	0.52 ± 0.09	0.55 ± 0.10	0.70 ± 0.13	0.501
Coixol	1.07 ± 0.36	1.16 ± 0.37	1.73 ± 0.49	0.510

Values reported as means ± standard error. Different letters denote statistical significance at *p* < 0.05. ^1^ GRASS: beef fed a diverse pasture; ^2^ GRAIN: beef fed a total mixed ration (TMR); ^3^ GRAPE: beef fed TMR + 5% DM grapeseed extract.

## Data Availability

The original contributions presented in the study are included in the article/Supplementary Materials. Further inquiries can be directed to the corresponding author.

## Supplementary Materials

The following supporting information can be downloaded at: https://www.mdpi.com/article/10.3390/foods12193547/s1, Table S1: Phytochemical profile of feeds (mg per 100 g); Beef Metabolomics Compounds Information.xlsx.
