# Supplementary material for: Measuring the Phytochemical Richness of Meat: Effects of Grass/Grain Finishing Systems and Grapeseed Extract Supplementation on the Fatty Acid and Phytochemical Content of Beef
Source: Foods. 2023 Sep 24;12(19):3547. doi: 10.3390/foods12193547 (PMC10572853; doi:10.3390/foods12193547)
Supplement: Supplementary file 1 [file foods-12-03547-s001.zip › Supplementary Table S1.pdf]

Supplementary Table S1. Phytochemical profile of feeds (mg per 100 g).

	GRAIN[1]	GRAPE[2]	*p*-Value
**Isocitric acid lactone**	6.44 ± 1.09	6.80 ± 1.48	0.850
**Sucrose**	32.07 ± 3.19	32.26 ± 5.91	0.978
**D-Maltose**	38.50 ± 3.90	37.58 ± 6.29	0.906
**Citric acid**	2.45 ± 0.31	2.01 ± 0.70	0.594
**D-Tartaric acid**	1.25 ± 0.07	1.17 ± 0.16	0.653
**Succinic acid**	3.86 ± 0.42	3.74 ± 0.43	0.848
**Adipic acid**	2.13 ± 0.54	2.30 ± 0.76	0.862
**(-)-Epicatechin gallate**	26.67 ± 25.00	188.13 ± 124.82	0.288
**Epicatechin**	2.43 ± 2.35	13.19 ± 7.57	0.254
**Naringenin**	0.10 ± 0.01	0.11 ± 0.02	0.688
**Hesperidin**	0.54 ± 0.09	0.43 ± 0.12	0.480
**Hesperetin**	0.14 ± 0.00	0.14 ± 0.01	0.674
**Apigenin**	0.23 ± 0.03	0.18 ± 0.06	0.514
**Catechol**	1.57 ± 0.08	1.57 ± 0.10	0.986
**Chrysin**	0.04 ± 0.00	0.05 ± 0.00	0.400
**Diosmetin**	0.60 ± 0.04	0.57 ± 0.02	0.424
**Isorhamnetin 3-rutinoside**	17.37 ± 3.18	12.49 ± 2.28	0.235
**Eriodictyol-7-o-glucoside**	0.11 ± 0.03	0.13 ± 0.06	0.832
**Isorhamnetin**	2.25 ± 0.50	1.71 ± 0.15	0.325
**Biochanin A**	0.01 ± 0.00	0.00 ± 0.00	0.945
**Kaempferol**	0.30 ± 0.05	0.51 ± 0.25	0.464
**Pyrogallol**	0.06 ± 0.01	0.05 ± 0.01	0.727
**3,4-Dihydroxybenzoic acid**	5.31 ± 0.39	5.43 ± 0.65	0.884
**Salicylic acid**	3.47 ± 0.13	3.16 ± 0.29	0.371
**Vanillic acid**	7.22 ± 0.28	6.34 ± 0.31	0.065
**2,6-Dihydroxybenzoic acid**	10.10 ± 0.97	12.57 ± 1.14	0.139

**Quercetin**	4.20 ± 0.35	6.95 ± 2.54	0.359
**Caffeic acid**	0.20 ± 0.02	0.16 ± 0.01	0.162
**Ellagic acid**	0.07 ± 0.07	1.92 ± 1.62	0.338
**Pyrocatechol sulfate**	3.34 ± 0.22	3.35 ± 0.57	0.990
**Syringic acid**	2.23 ± 0.10	2.18 ± 0.25	0.849
**Ethyl gallate**	0.01 ± 0.01	0.09 ± 0.04	0.265
**Chlorogenic acid**	48.52 ± 7.34	35.96 ± 3.67	0.150
**Coixol**	0.43 ± 0.11	0.28 ± 0.03	0.225
**3,5-Dihydroxybenzoic acid**	5.21 ± 0.31	5.75 ± 0.31	0.248
**Vanillin**	5.58 ± 0.55	6.74 ± 0.47	0.136
**4-Hydroxybenzoic acid**	6.81 ± 0.63	6.67 ± 0.94	0.913
**Gallocatechin gallate**	1.67 ± 0.38	4.42 ± 2.11	0.285
**Arbutin**	0.53 ± 0.04	0.50 ± 0.06	0.698
**p-Coumaric acid**	8.70 ± 0.30	8.01 ± 0.30	0.132
**Shikimic acid**	1.70 ± 0.13	1.59 ± 0.36	0.801
**Thiamine**	0.07 ± 0.00	0.07 ± 0.00	0.816
**Niacin**	0.06 ± 0.01	0.01 ± 0.00	< 0.010
**Nicotinic acid**	0.11 ± 0.00	0.14 ± 0.03	0.428
**Pyridoxine**	0.01 ± 0.00	0.01 ± 0.00	0.490
**Riboflavin**	1.41 ± 0.15	0.99 ± 0.13	0.062
**Alpha-tocopherol**	1.29 ± 0.14	1.19 ± 0.26	0.756
**Pantothenic acid**	0.16 ± 0.01	0.14 ± 0.00	0.096
**Stachydrine**	1.03 ± 0.10	1.05 ± 0.10	0.848
**Trigonelline**	0.11 ± 0.01	0.11 ± 0.01	0.686
**Tyramine**	0.00 ± 0.00	0.06 ± 0.06	0.420
**Betaine**	0.58 ± 0.03	0.44 ± 0.04	< 0.050
**Tricine**	0.31 ± 0.06	0.27 ± 0.09	0.701
**Tamarixetin**	1.74 ± 0.28	1.27 ± 0.20	0.203

**Sophoricoside**	6.54 ± 1.86	6.57 ± 3.10	0.995
**Quercetin-3-O-lactoside**	2.74 ± 0.27	5.03 ± 1.96	0.327
**Scopoletin**	0.02 ± 0.00	0.02 ± 0.00	0.835
**Calycosin**	0.07 ± 0.03	0.07 ± 0.05	0.981
**Glycitein**	0.35 ± 0.11	0.29 ± 0.13	0.762
**Naringenin-7-O-glucoside**	37.38 ± 7.68	31.60 ± 2.46	0.488
**Xanthohumol**	0.06 ± 0.02	0.03 ± 0.00	0.195
**Schaftoside**	2.89 ± 0.32	2.10 ± 0.46	0.209
**Chrysoeriol**	2.56 ± 0.22	2.33 ± 0.12	0.363
**Isovitexin/Nitexin**	6.60 ± 0.52	5.53 ± 1.15	0.438
**Jasmonic acid**	0.70 ± 0.09	0.51 ± 0.14	0.299
**Morin**	0.91 ± 0.04	0.89 ± 0.10	0.836

Values reported as means ± standard error. Results of two-sample t-test. Statistical significance at $p < 0.05$. [1]GRAIN: total mixed ration (TMR) ($n = 11$); [2]GRAPE: TMR + 5% DM grapeseed extracts ($n = 4$). N.B.: no GRASS (diverse pasture) samples were left for the phytochemical analysis.
